# Transcriptome sequencing of cochleae from constant-frequency and frequency-modulated echolocating bats

**DOI:** 10.1038/s41597-020-00686-w

**Published:** 2020-10-13

**Authors:** Lu Ma, Haijian Sun, Xiuguang Mao

**Affiliations:** 1 School of Ecological and Environmental Sciences, East China Normal University, Shanghai, 200062 China; 2 Institute of Eco-Chongming (IEC), East China Normal University, Shanghai, 200062 China; 3 Present Address: Changsha Central Hospital, University of South China, Changsha, 410011 China

**Keywords:** Evolutionary genetics, Ecological genetics, Comparative genomics

## Abstract

Echolocating bats are fascinating for their ability to ‘see’ the world in the darkness. Ultrahigh frequency hearing is essential for echolocation. In this study we collected cochlear tissues from constant-frequency (CF) bats (two subspecies of *Rhinolophus affinis*, Rhinolophidae) and frequency-modulated (FM) bats (*Myotis ricketti*, Vespertilionidae) and applied PacBio single-molecule real-time isoform sequencing (Iso-seq) technology to generate the full-length (FL) transcriptomes for the three taxa. In total of 10103, 9676 and 10504 non-redundant FL transcripts for *R. a. hainanus*, *R. a. himalayanus* and *Myotis ricketti* were obtained respectively. These data present a comprehensive list of transcripts involved in ultrahigh frequency hearing of echolocating bats including 26342 FL transcripts, 24833 of which are annotated by public databases. No further comparative analyses were performed on the current data in this study. This data can be reused to quantify gene or transcript expression, assess the level of alternative splicing, identify novel transcripts and improve genome annotation of bat species.

## Abstract (summary)

**Table:** 

Measurement(s)	cochlea • transcriptome • sequence feature annotation
Technology Type(s)	isoform sequencing • sequence annotation
Factor Type(s)	species
Sample Characteristic - Organism	Rhinolophus affinis • Myotis ricketti

Machine-accessible metadata file describing the reported data: 10.6084/m9.figshare.12999614

## Background & Summary

Most bats have evolved echolocation to navigate, explore environment and hunt prey in the darkness^1^. All echolocating bats require ultrahigh frequency hearing for reception of ultrahigh frequency sounds, which is essential in the process of echolocation^2^. High frequency hearing is also important for non-echolocating mammals, including human. However, the molecular mechanisms underlying the origin of high frequency hearing is still unknown^3^. Echolocating bats with ultrahigh frequency hearing provide a unique model for studying the molecular basis of high frequency hearing in mammals.

Modulation of gene expression and alternative mRNA splicing are two major forms of transcriptional regulation, responsible for the origin of novel phenotype and phenotypic diversity^4–7^. Recently, high-throughput transcriptome sequencing (RNA-seq) of cochlear tissue has been used to uncover differentially expressed genes possibly associated with the origin of ultrahigh frequency hearing^8^, the divergence of different echolocating types^9^ and echolocation call frequency variation^10^. In these earlier studies, the reference used for quantification of gene expression was from a *de novo* assembly based on the short RNA-seq reads which may contain many artificial transcripts^11^. The PacBio single-molecule real-time isoform sequencing (Iso-seq) can generate full-length (FL) sequences of all transcripts without the need for assembly^12^, which has been integrated with RNA-seq for transcriptome quantification in multiple studies^12,13^. PacBio Iso-seq is also used to detect alternative splicing events without the help of a reference genome sequence^14^ and to identify previously unannotated transcripts^15^. So far, no PacBio Iso-seq study has been conducted on the cochlear tissue of echolocating bats.

In this study we generated FL transcriptome datasets from the cochlear tissue of two kinds of echolocating bats using PacBio Iso-seq. Echolocating bats with ultrahigh frequency hearing (laryngeal echolocation) include constant-frequency (CF) bats and frequency-modulated (FM) bats^16^. We collected cochlear tissues from both CF and FM bats in order to get a comprehensive list of transcripts involved in ultrahigh frequency hearing (Table 1). We chose *Rhinolophus affinis* (Rhinolophidae) and *Myotis ricketti* (Vespertilionidae) as the representatives for CF and FM bats, respectively. To investigate the genetic basis of intraspecific echolocation call frequency variation in future, we included two *Rhinolophus affinis* subspecies (*R. a. hainanus* and *R. a. himalayanus*) which show divergent echolocation call frequencies^17,18^. For clarity, the FL transcriptomes from the CF bats (*R. a. hainanus* and *R. a. himalayanus*) and FM bat (*Myotis ricketti*) were called FL-CF-Rhai, FL-CF-Rhim and FL-FM-Myo, respectively. After PacBio Iso-seq data processing, we obtained a total of 10103, 9676 and 10504 non-redundant FL transcripts for FL-CF-Rhai, FL-CF-Rhim and FL-FM-Myo respectively, ranging in size from 201 bp to 9740 bp (Table 2). The number of transcripts annotated in NCBI non-redundant protein sequences (Nr) and the UniprotKB database at least once is 9564, 9079 and 10090, respectively (Table 3). By combining the datasets from the three taxa we also generated a FL transcriptome of echolocating bats (FL-CF-FM) which contains 26342 FL transcripts with 24833 of them annotated in Nr or UniprotKB database (Tables 2 and 3).

**Table 1:** Detailed information about Iso-seq libraries.

Sample	Tissue	OD260/280	OD260/230	28 S/18 S	Completeness (RIN)	SRA IDs	TSA IDs
FL-CF-Rhai	cochleae	2.13	1.8	1	7	SRR12062845	GIRV00000000
FL-CF-Rhim	cochleae	2.13	1.81	0.9	7	SRR12062844	GIRW00000000
FL-FM-Myo	cochleae	2.16	1.86	1.3	7.6	SRR12062843	GIRX00000000

**Table 2:** Statistics of the four FL transcriptomes generated in this study.

Sample	FL-CF-Rhai	FL-CF-Rhim	FL-FM-Myo	FL-CF-FM
Subreads number	3444947	3255638	3403451	
Total base (bp)	6448987299	6504282447	7190237257	
Mean length (bp)	1872	1998	2113	
**Classify**				
CCS number	137159	137160	152251	
Mean CCS read length (bp)	2443	2628	2732	
Number of Passes (mean)	22	20	19	
Reads with 5 and 3 Primers (in percent)	112912 (82.32%)	107080 (78.07%)	123700 (81.25%)	
Non-Concatamer reads with 5 and 3 Primers	111976 (81.64%)	105919 (77.22%)	122411 (80.4%)	
FLNC (Non-Concatamer Reads with 5 and 3 Primers and Poly-A Tail)	111806 (81.52%)	105713 (77.07%)	122222 (80.28%)	
**Arrow correction**				
Number of transcripts	10384	9984	10932	31300
Number of non-redundant transcripts	10103	9676	10504	26342
Total base (bp)	22746072	22932622	26578852	63358581
Mean length (bp)	2251	2370	2530	2405

**Table 3:** Annotation statistics for each of the four FL transcriptomes.

Database	FL-CF-Rhai	FL-CF-Rhim	FL-FM-Myo	FL-CF-FM
Nr	9555 (94.58%)	9069 (93.73%)	10067 (95.84%)	24793 (94.12%)
UniProt	9324 (92.29%)	8825 (91.21%)	9894 (94.19%)	24198 (91.86%)
At least one annotation	9564 (94.66%)	9079 (93.83%)	10090 (96.06%)	24833 (94.27%)

One limitation of this study is that we did not include biological replicates when generating the Iso-seq dataset for each taxon due to limited tissues available and a large amount of RNA required in PacBio Iso-seq library construction. Currently, the high cost for PacBio sequencing is another constraint to be considered. If the main aim of the study is to identify transcripts expressed in one or multiple tissues, as in most of current studies using FL transcriptome sequencing, it is unnecessary to include additional biological replicates. However, we pooled RNA from three individuals during library constructions of each of three echolocating bats in this study. By this way, we tried to avoid missing any transcripts due to degradation of RNA a specific individual and thus obtained a comprehensive list of transcripts expressed in cochlea.

The current FL transcriptomes generated in this study are sufficient to be reused in the several aspects. They can be used as the reference to reanalyze the RNA-seq datasets of cochlea in previous comparative transcriptomic studies^8–10^. Quantification of transcript expression by mapping reads to the FL transcriptome will help to improve the accuracy of identifying differentially expressed transcripts^12^. Moreover, by comparing with transcripts expressed in non-echolocating mammals, the current FL transcriptomes from echolocating bats will help to test whether alternative splicing plays an important role in the origin of novel phenotype (ultrahigh frequency hearing). In addition, FL transcriptomes from FM bats and two CF subspecies could be used to test the roles of alternative splicing in the divergence of different echolocating types (CF and FM) and in intraspecific echolocation call frequency variation. Finally, these FL transcriptome datasets will be useful for identification of novel transcripts and for improvement of genome annotation of *Rhinolophus affinis*,*Myotis ricketti*, and other bat species^19,20^.

## Methods

### Sample collection and RNA preparation

We captured nine adult male bats from China including three *Myotis ricketti* from Jiangsu on April 19, 2018, three *Rhinolophus affinis hainanus* from Hainan on May 6, 2019, and three *R. a. himalayanus* from Anhui on January 4, 2019. Bats were rapidly euthanized by cervical dislocation, and cochleae were collected and transferred to RNase-free PCR tubes. Tissue samples were frozen immediately in liquid nitrogen and stored at −80 °C until RNA extraction. All sampling procedures were in accordance with the guidelines of Regulations for the Administration of Laboratory Animals approved by the Animal Ethics Committee of East China Normal University (ID no: bf20190301).

**Fig. 1:**
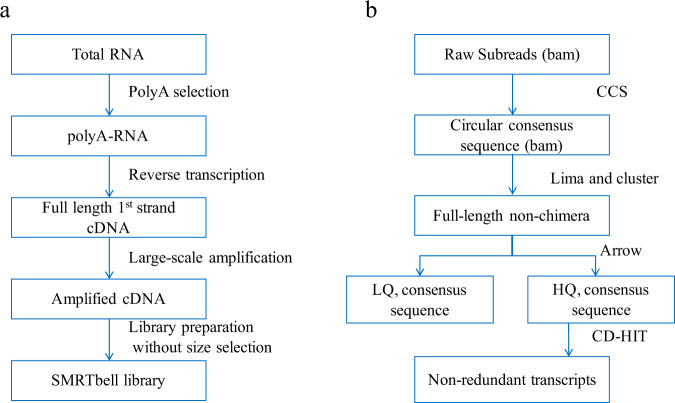
Overview of the sequencing data collection (**a**) and analysis pipeline (**b**).

RNA from each tissue was extracted individually using Trizol reagent (Invitrogen, CA, USA) according to the manufacturer’s instructions. Poly-A mRNAs were harvested using oligo-dT attached magnetic beads. RNA concentration was assessed using a NanoDrop spectrophotometer (Thermo Fisher Scientific, Waltham, USA), and RNA integrity number (RIN) values were assessed using an Agilent 2100 Bioanalyzer (Agilent Technologies, Santa Clara, USA) (Fig. 1 and Table 1).

### Library construction and full-length sequencing

RNA from three individuals of each taxon (*R. a. hainanus*, *R. a. himalayanus* and *Myotis ricketti*) were pooled to obtain enough amount of RNA (800–1000 ng) for PacBio Iso-seq library construction. We built one independent SMRTbell library for each taxon (a total of three libraries) with the PacBio DNA Template Prep Kit 3.0 according to the manufacturer’s instructions. SMRT sequencing was performed with the PacBio Sequel platform.

### Generation of the full-length transcriptomes

PacBio Iso-seq raw data (subreads) from each taxon were analyzed using the SMRTLink software (v6.0). First, the circular consensus sequences (CCSs) were generated from subreads. The FL sequences with intact 5′ and 3′ primers and poly-A tails were identified and used in the following analysis. Then, lima, implemented in IsoSeq. 3 from SMRTLink, was used to remove primers and identify barcodes. After trimming the poly-A tails and chimeric, cluster function in IsoSeq. 3 was used to produce full-length non-chimeric (FLNC) sequences. FLNC sequences were polished with arrow model in IsoSeq. 3 to generate high quality isoforms with an accuracy >99%. Redundancy was removed using CD-HIT-EST (version 4.7)^21^ with 99% sequence similarity threshold and transcripts shorter than 200 bp were filtered, resulting in a FL transcriptome (Fig. 1). Finally, by combining the three FL transcriptomes and removing redundant transcripts, we generated a FL transcriptome from both CF and FM bats (hereafter called FL-CF-FM). We assessed the completeness of each of the four FL transcriptomes by searching against single-copy orthologues (4,104 genes shared by 50 mammal species; http://busco.ezlab.org) using mammalia_odb9 BUSCO version 3.0.2^22^.

### Functional annotation

Each of the four FL transcriptomes was functionally annotated by performing a local BLASTx search against two protein databases, the Nr protein database (http://www.ncbi.nlm.nih.gov, accessed December 1, 2019) and UniProtKB (http://www.expasy.ch/sprot, accessed July 6, 2019), with an E-value of 1e-5.

## Data Records

The raw FL sequencing data for each taxon have been deposited in the NCBI Sequence Read Archive (SRA) (Accession numbers: SRR12062845^23^, SRR12062844^24^ and SRR12062843^25^) (Table 1). The three FL transcriptomes from each of the three taxon have been deposited in the NCBI Transcriptome Shotgun Assembly (TSA) database (Accession numbers: GIRV00000000^26^, GIRW00000000^27^ and GIRX00000000^28^) (Table 1). The FL transcriptomes and functional annotation results for each of the four FL transcriptomes have been deposited in Figshare^29^.

## Technical Validation

### Quality control of the full-length transcriptomes

The FL transcriptomes for *R. a. hainanus*, *R. a. himalayanus* and *Myotis ricketti* were constructed based on sequencing data of three separated libraries on the PacBio Sequel platform. Specifically, a total of 3,444,947 subreads with 6,448,987,299 nucleotides, 3,255,638 subreads with 6,504,282,447 nucleotides and 3,403,451 subreads with 7,190,237,257 nucleotides were generated for *R. a. hainanus*, *R. a. himalayanus* and *Myotis ricketti* respectively. After quality control, we obtained 137,159 circular consensus sequencing (CCS) reads for *R. a. hainanus*, 137,160 CCS reads for *R. a. himalayanus* and 152,251 CCS reads for *Myotis ricketti*. With the standard IsoSeq. 3 classification and clustering pipeline, we identified 111,806 FLNC for *R. a. hainanus*, 105,713 FLNC for *R. a. himalayanus* and 122,222 FLNC for *Myotis ricketti*. After isoform-level polishing, 10384, 9984 and 10932 high quality isoforms were retained in *R. a. hainanus*, *R. a. himalayanus* and *Myotis ricketti* respectively. After removing redundancy with CD-HIT-EST and filtering isoforms shorter than 200 bp, the final FL transcriptomes for *R. a. hainanus*, *R. a. himalayanus* and *Myotis ricketti* (FL-CF-Rhai, FL-CF-Rhim and FL-FM-Myo, respectively) contain 10103, 9676 and 10504 FL isoforms with an average length of 2251, 2370 and 2530 bp, respectively (Table 2). Finally, the FL transcriptome from both CF and FM bats (FL-CF-FM) contains 26,342 transcripts with an average length of 2,405 bp (Table 2). BUSCO analysis revealed that a total of 2,354 (57.4%) BUSCOs were included in FL-CF-FM. We also found 39.9%, 38.1% and 41.9% BUSCOs in FL-CF-Rhai, FL-CF-Rhim and FL-FM-Myo, respectively (Table 4). Given the highly specialized function of the cochlea, we should not expect a high level of BUSCO value in FL transcriptome of cochlea. A recent single cell RNA-seq study has identified a similar number of genes expressed in the murine cochlea (a total of 12,944)^30^.

**Table 4:** Completeness of each of the four FL transcriptomes assessed by benchmarking universal single-copy ortholog (BUSCO) analysis.

	FL-CF-Rhai	FL-CF-Rhim	FL-FM-Myo	FL-CF-FM
Complete BUSCOs (C)	1458 (35.5%)	1363 (33.2%)	1526 (37.2%)	2122 (51.7%)
Complete and single-copy BUSCOs (S)	1082 (26.4%)	997 (24.3%)	1053 (25.7%)	917 (22.3%)
Complete and duplicated BUSCOs (D)	376 (9.2%)	366 (8.9%)	473 (11.5%)	1205 (29.4%)
Fragmented BUSCOs (F)	179 (4.4%)	202 (4.9%)	194 (4.7%)	232 (5.7%)
Missing BUSCOs (M)	2467 (60.1%)	2539 (61.9%)	2384 (58.1%)	1750 (42.6%)
Total BUSCO groups searched	4104 (100.0%)	4104 (100.0%)	4104 (100.0%)	4104 (100.0%)

### Quality control of annotation

Four FL transcriptomes (FL-CF-Rhai, FL-CF-Rhim, FL-FM-Myo, and FL-CF-FM) were functionally annotated by performing DIAMOND and BLASTx searches against the Nr and UniProt databases separately. For FL-CF-FM, 24,793 and 24,198 transcripts were annotated by Nr database and UniProt database, respectively (Table 3). After combining the annotation results from the two databases, a total of 24,833 transcripts were annotated in at least one database. We obtained similar annotation results for FL-CF-Rhai, FL-CF-Rhim and FL-FM-Myo (Table 3). Transcripts without annotations might be novel isoforms of echolocating animals or due to the lack of representative sequences for cochlea in public databases.

## Data Availability

The software versions and parameters used in this study are described below.1.SMRTlink: version 6.0, parameters: pbccs.task_options.max_length = 20000 pbccs.task_options.min_length = 300.2.CD-Hit-Est: version 4.7, parameters: -c 0.99 -T 20 -G 0 -aL 0.90 -AL 100 -aS 0.98 -AS 30 -M 0 -d 0.3.BUSCO: version 3.0.2, default parameters. -m tran -e 1e-05.4.BLASTx: version 2.2.29+, parameters: -outfmt 6, -e value 1e-5 --max-target-seqs 1.5.DIAMOND: version 0.9.24.125.
